# Salmagundi

**Published:** 1888-01

**Authors:** 


### SALMAGUNDI.

EDITED BY E. G. BETTY, D.D.S.

WE are informed that a new form of chromic acid, prepared by Merck, and alleged to be absolutely free from sulphuric acid, and hence very slowly deliquescent in dry air, is now procurable. The acid is in the form of small, dry, red crystals, readily soluble in water and in alcohol. It is said that the acid in this form does not spread over the surrounding tissue, and is therefore better suited for use in places where it would be dangerous to employ the ordinary acid.

Cleveland Dental Society.—A recent election of officers resulted in the choice of the following gentlemen to serve for the ensuing year: Pres. Ira Brown; Vice Pres. J. R. Owens; Sec. Jere Robinson; Treas. S. B. Dewey.

The retiring President, D. R. Jennings, read an interesting address in which he suggested that the society hold clinics at its meetings, in addition to the reading of papers and discussions. The idea is a good one and will be productive of much good. Though a very young Society, Cleveland means business, and we wish to hear more from that neck of the woods.

---

Dr. N. S. Hoff, of Cincinnati, has given up his business in the city and associated himself with the corps of instructors in the Dental Department of the University of Michigan. He goes as a teacher in the operative department, where his unusual ability as an executant will soon make itself felt in the quality of instruction he is capable of imparting to those under his supervision. His individuality as an operator is recognized by a large number in the profession, and the Board of Regents is to be congratulated upon securing for the university the services of so competent a man. The best wishes of a large circle of friends attend him.

---

English as Written.—In the October number of the *Ohio Journal,* the editor undertakes to teach us a little English and his idea is good, provided he would practice what he preaches, *i. e;* study his dictionary a little before he assumes the double role of critic and teacher. We will name two instances in which he has overreached himself, from excess of zeal probably.

On page 492, he quotes the following sentence from a paper read by a schoolteacher: "We were only counted for what we were worth" and then remarks, "Only *counted*? Were they not estimated? regarded? held for what they were worth?"

If our friend of *The Journal* will take the trouble to consult Webster or Stormonth he will find that the schoolteacher is right and the critic did not know it. Again; on the same page, the next sentence in fact, he blunders a second time in this wise:

"And so we could go on for a dozen pages, *selecting from only standard writers*" (italics ours). In this case he misconceives the meaning of the word "only" in its relation to the rest of the sentence, it being not as he supposes an adjective, but an adverb. His sentence should read "selecting only from standard writers" or "selecting from standard writers only," both of which constructions place the adverb in its proper position to qualify the active verb "selecting." Besides this, our critic does not seem disposed to allow words much latitude of meaning, forgetting that custom does more to make or break the sense of a word or phrase, than all the pedagogues combined.

